# Management of Choledochal Cysts at a Tertiary Care Centre: A Nine-Year Experience from India

**DOI:** 10.1155/2020/8017460

**Published:** 2020-04-20

**Authors:** Pranav Honnavara Srinivasan, Amudhan Anbalagan, Rajendran Shanmugasundaram, Naganathbabu Obla Lakshmanamoorthy

**Affiliations:** Institute of Surgical Gastroenterology, Rajiv Gandhi Government General Hospital, Madras Medical College, Chennai, India

## Abstract

**Background:**

Although choledochal cyst disease is seen predominantly in childhood, it is becomingly increasingly diagnosed in adult patients.

**Methods:**

Data of 36 patients with choledochal cysts managed in our institute between January 2010 and December 2018 were retrospectively analyzed.

**Results:**

Median age at presentation was 37 years (range: 13–72 years). Female-to-male ratio was 3.5 : 1. All patients were symptomatic, and abdominal pain was the most common symptom. 72.2% had other associated conditions. There was a considerable delay from the onset of symptoms to referral, median duration being 348 days. There were 28 cases of type I (77.8%), 5 cases of type IVA (13.9%), and 3 cases of type IVB (8.3%). Cyst excision with Roux-en-Y hepaticojejunostomy was performed in 29 (80.55%) cases. This procedure was combined with a left lateral sectionectomy, left hepatectomy, and radical cholecystectomy in 1, 2, and 1 cases, respectively. Lilly's technique was used in 2 cases, and cyst excision with hepaticoduodenostomy was performed in 1 case. Early complications were seen in 21 patients (58.3%), and late complications were seen in 5 patients (13.8%). 2 patients were found to have associated malignancies. One patient was detected to have cholangiocarcinoma in the resected liver incidentally, and another patient was diagnosed to have gall bladder cancer intraoperatively.

**Conclusion:**

Choledochal cysts should be considered in the differential diagnosis of adults presenting with epigastric or right hypochondrium pain or jaundice. A thorough preoperative evaluation is required. Cyst excision with Roux-en-Y hepaticojejunostomy forms the standard treatment in most cases. Long-term follow-up is essential for management of complications and early detection of malignant change.

## 1. Introduction

Choledochal cysts (CCs) are rare pathological congenital dilatations of the biliary tract. Vater and Ezler first reported these entities in 1723 [1]. The term “choledochal cyst” is a misnomer as the dilatation may involve the entire biliary tract and not restricted to the choledochus (common bile duct) alone. Hence, more apt terms such as “biliary cysts” or “bile duct cysts” should be used. Because of the long-term usage of the term “choledochal cyst,” the usage of this term has persisted and is still accepted.

CCs are predominantly a disease of children with 80% of these cysts diagnosed within the first decade of life [2–4]. Presentation in adults accounts for about 20% of the cases [5, 6]. There has been an increasing trend in the number of adult cases due to increasing awareness of this rare disease as well as better accuracy of imaging studies [7, 8].

CCs have mostly been reported from East Asian countries where they have a high incidence of about 1 : 1000 [9]. Data on incidence in the Indian subcontinent are inadequate.

The most widely used classification for CC is the Todani modification (1977) of the Alonso-Lej classification [10, 11] which divides the cysts into 8 alphanumerical subtypes. Treatment of CC includes excision of the cyst and bilioenteric anastomosis in most cases.

We present our experience in the management of CC in this retrospective study.

## 2. Materials and Methods

Between 1^st^ January 2010 and December 31^st^ 2018, 36 patients with CC were treated surgically at the Institute of Surgical Gastroenterology, Rajiv Gandhi Government General Hospital, Chennai, India. The patient data were analyzed retrospectively from our prospectively maintained database. Patient demographics, symptoms at presentation, blood and imaging investigations, previous treatment, operative outcomes, and follow-up data were analyzed. All imaging studies were retrospectively reviewed again from our imaging database specially to confirm the presence or absence of anomalous pancreaticobiliary duct union (APBDU) to alleviate reporting bias. In all cases in this study, the diagnosis was confirmed by histopathological examination of the surgical specimen. Follow-up was done every 3 months after surgery for 2 years followed by yearly follow-up thereafter. A physical examination, ultrasound of the abdomen, and liver function tests were performed at every follow-up visit. Data regarding follow-up were obtained through outpatient records and through telephonic communication. Long-term follow-up data were available in 31 patients.

Cysts were classified according to the Todani classification, and the complications were defined as early if they occurred within 30 days and late if they occurred after 30 days. Both early and late complications were classified using the Clavien–Dindo classification.

## 3. Results

### 3.1. Demographics

**Figure 1:**
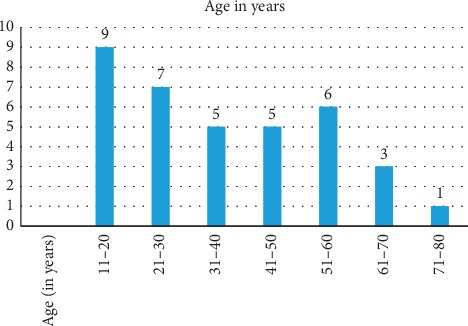
Age in years.

A total of 36 patients were included in this retrospective study. A female predominance was observed in our series, with 28 females and 8 males, the ratio being 3.5 : 1. Median age was 37 years, the youngest patient being 13 years and the oldest, 72 years. The decadewise distribution of our patients is shown in Figure 1.

### 3.2. Symptomatology

None of the CCs were detected incidentally, and all presented with at least 1 symptom. 97.2% (*n* = 35) had either a right upper abdominal or epigastric pain. 25% (*n* = 9) had anorexia, 19.4% (*n* = 7) had loss of weight, and 16.7% (*n* = 6) had jaundice. 13.8% (*n* = 5) had cholangitis, and 5.5% (*n* = 2) patients had pancreatitis. A mass was palpable clinically in 16.7% (*n* = 6).

The classical triad of symptoms of abdominal pain, jaundice, and mass was detected only in 11.1% (*n* = 4).

### 3.3. Blood Investigations

Total serum bilirubin was elevated in 25% (*n* = 9) with a mean of 2.14 mg/dL (range 0.2–15 mg/dL). Serum alkaline phosphatase was elevated in a total of 30.5% (*n* = 11) with a mean of 129.9 IU/L (range 13–450).

### 3.4. Imaging

Transabdominal ultrasound was used as the initial investigation in all cases and could reliably diagnose the cyst in 58.3% (*n* = 21). A cross-sectional imaging study was used as the main imaging modality to identify the type and location of cysts and to identify other associated pathology such as stones, pancreatitis, and malignancy. Magnetic resonance cholangiopancreatography (MRCP) was used for diagnosis in 86% (*n* = 31), computed tomography (CT) in 2.7% (*n* = 1), and both MRCP and CT were used in the diagnosis in 11.1% (*n* = 4). APBDU was detected in 3 patients (8.33%) on preoperative imaging. Hepatobiliary iminodiacetic acid scan (HIDA) was not performed on any of the patients. The frequency of distribution of the symptoms and associated conditions in relation to the type of cysts is demonstrated in Table 1.

### 3.5. Rate of Misdiagnosis before Referral

27.8% (*n* = 10) cases were misdiagnosed before referral to our center. Six patients were misdiagnosed as choledocholithiasis and one each as liver abscess, distal bile duct stricture, duodenal diverticulum, and acute cholecystitis.

### 3.6. Associated Conditions

72.2% (*n* = 26) had associated conditions along with the cyst. Stones were present in the biliary tract in 36.1% (*n* = 13) in which 11.1% (*n* = 4) patients had cystolithiasis (stones in the CC) only, 16.7% (*n* = 6) patients had cystolithiasis along with cholelithiasis, and 8.33% (*n* = 3) had cystolithiasis with hepatolithiasis (intrahepatic calculi). 13.8% (*n* = 5) patients had evidence of distal biliary stricture, and there were two cases each having perivaterian diverticula, liver abscess, and chronic pancreatitis. Pancreatic divisum and nonrotation of intestine were present in one case each.

### 3.7. Duration from Onset of Symptoms to Referral

The median duration from the onset of the first symptom till referral varied from 125 days to 640 days (median = 348 days).

### 3.8. Therapy before Referral

22.2% (*n* = 8) patients underwent endoscopic or surgical treatment before referral. ERCP and biliary stenting was performed in 13.8% (*n* = 5) patients, cholecystectomy was performed in one case with type IVA cyst, cholecystectomy with CBD exploration was performed for stone removal in one case, and laparotomy and drainage was performed in one case with a coexisting liver abscess.

### 3.9. Cyst Types

Cysts were classified according to the Todani modification of the Alonso-Lej classification illustrated in Table 2.

There were no cases of type II, III, or V in our study.

There were 28 cases of type I (77.8%), 5 cases of type IVA (13.9%), and 3 cases of type IVB (8.3%).

### 3.10. Surgical Procedures

Types of surgical procedures performed are depicted in Table 3. All cases were performed by open procedure.

One patient underwent radical cholecystectomy in addition to cyst excision due to intraoperative suspicion of gall bladder cancer. Two patients with monolobar Caroli's disease underwent left hepatectomy as an additional procedure, and histopathological examination in one of these patients showed an incidental intrahepatic cholangiocarcinoma.

### 3.11. Complications

There was no mortality in our study. All the complications were classified according to the Clavien–Dindo classification.

### 3.12. Early Complications

  21/36 patients had complications—58.3% morbidity.
  18 patients had grade I complications, of which 14 had wound infection, and 4 had bile leak which were managed conservatively.
  1 had a grade II complication— pancreatic fistula requiring parental nutrition.
  2 patients had grade IIIB complications—1 patient with wound dehiscence requiring secondary suturing and another with biliary peritonitis requiring laparotomy and lavage.
  There were no grade IIIA, IV, or V complications. The frequency of distribution of early complications is shown in Table 4.

### 3.13. Late Complications

  5/36–13.8% rate of long-term morbidity.
  3 cases developed grade II complications—adhesive intestinal obstruction but responded to conservative management.
  2 patients developed grade IIIB complications. One case who developed pancreatic fistula as an early complication developed recurrent episodes of pancreatitis, which resolved after endoscopic pancreatic stenting. Another patient with Roux-en-Y hepaticojejunostomy developed stricture at the anastomotic site 28 months after the initial surgery and required surgical revision of the anastomosis. The frequency of distribution of late complications is shown in Table 5.

### 3.14. Malignancy

  Malignant tumors were detected to be 5.5 % (*n* = 2) in this study.
  One case aged 46 years with type I cyst had suspicion of gall bladder thickening detected intraoperatively for whom an intraoperative decision to perform a radical cholecystectomy was taken. Subsequent histopathological examination of the specimen showed gall bladder carcinoma with evidence of infiltration into the muscularis. This patient is still alive without recurrent disease on follow-up 4 years following surgery.
  One patient aged 67 years with type IVA cyst with monolobar disease who underwent left hepatectomy with cyst excision was incidentally detected to have an intrahepatic cholangiocarcinoma on evaluation of the pathological specimen. This patient is alive and without recurrence 40 months after the surgery.
  8.33% (n = 3) were detected to have mild dysplasia in their CC specimens.
  No malignancies of the biliary tract were detected during the follow-up period in any of the patients.

### 3.15. Cyst Size

Cyst size denoted by the largest diameter measured on the pathological specimen varied from 1.6–9.7 cm with a median cyst size of 3.5 cm.

### 3.16. Follow-Up

The follow-up period ranged from 125 days to 2,796 days with a median of 922 days. 5 patients were subsequently lost to follow-up.

## 4. Discussion

Choledochal cysts (CCs) are a wide spectrum of disorders encompassing various types of pathological cystic dilatations of the biliary tree [6].

The incidence of CC is higher in Japan and Korea (1 : 1000 live births) compared with Western countries (1 : 100000–1 : 150000 live births) [9, 12]. The incidence in the Indian subcontinent is not reliably known due to paucity of data on CC from this part of the world. Most studies show a female predominance consistent with other biliary system disorders, and the female-to-male ratio varies from 3 : 1 to 4 : 1 [13–16].

CCs are mainly disorders of children with majority being diagnosed in the first decade of life. Adult presentations form about 20% of the total [5, 6]. However, there appears to be a rise in the number of adult patients being diagnosed and this is hypothesized to be due to a variety of factors including institutional referral biases, a rising awareness about the disease and increasing number and accuracy of cross-sectional imaging studies being performed [7, 8, 17–19].

The presentation in adults differs from that of children, in that adults have more severe acute biliary or pancreatic symptoms as the cyst disease is present for a relatively longer duration [7]. However, in children, the most common symptom is jaundice, and in adults, it is abdominal pain [16]. The classical triad of right upper quadrant pain, palpable mass, and jaundice manifests in about 85% of children when compared with only 25% in adults [20]. In our study, the classical triad was seen in 11.1%.

The most widely used classification system for CC is the Todani modification of the Alonso-Lej classification which classifies cysts based on cholangiographic anatomy and the extent of involvement of biliary system (Table 2) [10, 11].

The exact etiology of CC is unknown, and various hypotheses have been proposed. The most widely accepted hypothesis is the common channel theory with anomalous pancreaticobiliary duct union (APBDU) proposed by Babbitt in 1969 [21]. This hypothesis postulates that the junction of the pancreatic and biliary ducts is abnormally positioned outside the duodenal wall instead of the normal intramural location in the duodenal wall. The union of these ducts forms a single duct with a long common channel (>15 mm) proximal to the regulatory duodenal sphincter complex. This leads to reflux of the proteolytic enzyme-rich pancreatic secretions into the biliary system, causing weakening of the wall of the biliary system with eventual cystic dilatation. This hypothesis is supported by many studies which found a high prevalence of long common channel (68–94%) in patients with CC [20]. However, this hypothesis fails to explain in the context of predominant intrahepatic cystic disease with absent or minimal extrahepatic involvement. Furthermore, many other studies have reported APBDU with a much lower prevalence (14–44%) [22, 23]. This has resulted in many other hypotheses given due consideration.

The second hypothesis is that of presence of oligoganglionosis of the biliary tree, leading to inadequate autonomic innervation of the biliary tract. Dysmotility then results with functional obstruction and resultant proximal dilatation. This pathophysiology is similar to that of esophageal achalasia cardia and colonic Hirschsprung disease [5].

The third hypothesis is that partial biliary obstruction in patients results in higher proximal ductal pressures with eventual dilatation [20].

Another hypothesis is that of sphincter of Oddi dysfunction, where a tonically contracted sphincter complex predisposes to reflux of pancreatic secretions into the biliary tract leading to weakening of the ductal walls and subsequent cystic dilatation [24].

None of the above hypotheses is unequivocally accepted universally. Presumably, many factors contribute to varying extents to the development of the disease. In our study, we observed the presence of APBDU in only 8.33%. We postulate that oligoganglionosis, partial biliary obstruction, and sphincter of Oddi dysfunction contributed to a more significant role in the pathogenesis of CC than APBDU in our cases.

Ultrasonography is often used as the initial imaging investigation due to its wide availability, lower cost, and ease of performance. However, it lacks sensitivity due to many factors including lack of awareness among radiologists regarding CC, operator dependent nature of ultrasonography, hindrance of adequate visualization of the cyst by duodenal gas, and difficulty in differentiating CC from a distended gall bladder from cholecystitis [20].

**Figure 2:**
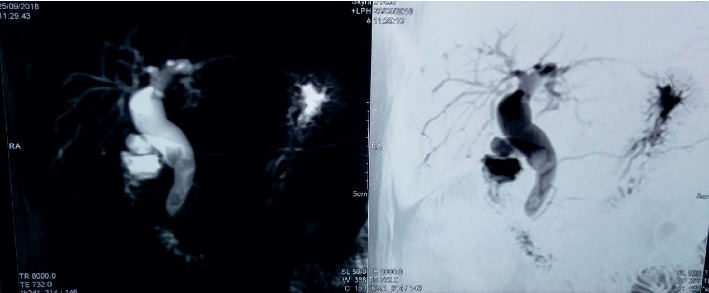
Type 1 choledochal cyst with multiple stones visible in the cyst.

Magnetic resonance cholangiopancreatography (MRCP) is preferred over computed tomography (CT) for preoperative diagnosis and is the current gold standard as it does not involve radiation exposure and defines the biliary system anatomy superiorly [20]. Cyst size, location, number, and presence of APBDU are accurately demonstrated by MRCP. Additionally, it can be performed in patients with renal disorders in whom contrast administration presents a risk or potentiating renal injury. Although endoscopic retrograde cholangiopancreatography (ERCP) has been replaced by MRCP for diagnosis, it is still being performed in regions where MRCP facilities are unavailable. ERCP has a role in cholangitis where it is used for drainage and in cholangiocarcinoma for performing brush biopsies [20].  Figure 2 illustrates a type 1 choledochal cyst with multiple calculi seen within the cyst.

Few authors prefer preoperative percutaneous transhepatic cholangiography in intrahepatic disease as it facilitates in planning liver resections [20].

In underdeveloped and developing countries, there is often a delay in diagnosis, and patients suffer from prolonged duration. Many factors are responsible for this delay including patient apathy, lack of awareness among clinicians about the disease in view of its rarity, scarcity of imaging facilities, and dearth of hepatopancreatobiliary specialists.

In addition, many patients tend to undergo unsatisfactory procedures, and the subsequent definitive surgery is considered to be more difficult due to violation of the anatomy of the right upper quadrant. In our study, the median duration from the onset of the first symptom to referral was 348 days, and 27.8% of cases were referred with incorrect diagnoses. Additionally, 22.2% of patients underwent suboptimal surgical or endoscopic procedures before eventual referral to our clinic. Hence, most patients continued to suffer from their symptoms for almost a year till a definitive diagnosis of CC disease was made. This echoes the findings of another study [22].

Untreated CCs are associated with an increased risk of complications, and hence the general principle is that CC should be excised whenever diagnosed. Associated complications include biliary stasis leading onto stone formation and recurrent episodes of cholangitis and pancreatitis due to biliary reflux into the pancreatic ductal system and protein plug impaction [20]. However, the most feared complication is the development of malignancy in the biliary tract. Patients with CC are reported to have a 6–30% chance of developing biliary tract malignancy in comparison with the low prevalence of cholangiocarcinoma in the general population (incidence of about 0.8/100000 persons in the USA) [25] the risk of biliary tract malignancy increases with age. In one study in which the overall incidence of biliary tract malignancy was 7.5%, children less than 18 years of age had 0.4% incidence, and those aged more than 18 years of age had an 11% incidence. In adults more than 60 years, the incidence was as high as 38%. Of those who developed biliary tract malignancy, 70% developed cholangiocarcinoma and 24% developed gall bladder cancer [26]. In our study, 13.83% had neoplastic changes with either malignancy (5.5%) or dysplasia (8.33%) detected on histopathological examination. It is probable that the risk of malignancy in choledochal cysts is underestimated in the current literature. One patient was diagnosed to have intrahepatic cholangiocarcinoma incidentally on pathological examination, and another patient who in addition to cyst excision underwent radical cholecystectomy based on an intraoperative finding of suspicious gall bladder wall thickening was diagnosed to have gall bladder carcinoma with infiltration of the muscularis. Both patients are alive and recurrence-free on long-term follow-up. The risk of malignant transformation is highest with type I and IV cysts, lesser in type V, and is the least in type II and III cysts.

Katabi et al. examined 36 histopathological specimens and found evidence of metaplasia in 40%, biliary intraepithelial neoplasia (BIN) in 28.5%, and invasive malignancy in 14.3%. They concluded that tumor progression in CC occurs in a metaplasia to neoplasia to carcinoma sequence [27]. In our study, 8.3% showed evidence of mild dysplasia.

### 4.1. Surgical Treatment

**Figure 3:**
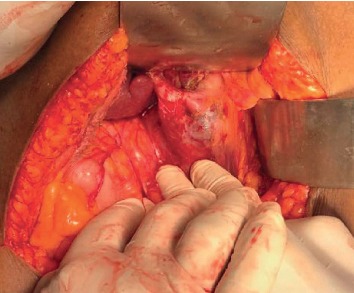
Intraoperative photo of the choledochal cyst depicted in the previous MRCP image. Note the large cyst emerging from the liver hilum extending to the duodenum.

**Figure 4:**
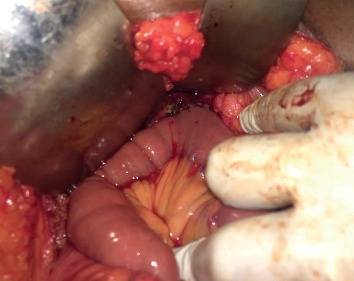
Intraoperative photo taken after choledochal cyst excision and completion of Roux-en-Y hepaticojejunostomy.

Historically, most patients with CC underwent internal drainage procedures. However, current standard of care entails complete excision of the affected biliary tract with reconstruction by a bilioenteric anastomosis in order to reduce potential long-term complications [20]. Biliary continuity may be established either by a Roux-en-Y hepaticojejunostomy or hepaticoduodenostomy [20, 28]. Although hepaticoduodenostomy is easier to perform, it is associated with an increased rate of complications (33%) when compared to Roux-en-Y hepaticojejunostomy. This includes duodenogastric reflux cholangitis and bile reflux gastritis. Reflux cholangitis may predispose to the development of cholangiocarcinoma and biliary reflux gastritis to gastric cancer. Hence, most centers currently perform biliary reconstruction by Roux-en-Y hepaticojejunostomy [20, 28].  Figure 3 is an intraoperative photograph of the same cyst illustrated in Figure 2 before excision, and Figure 4 is the intraoperative photograph after excision of the cyst and completion of Roux-en-Y hepaticojejunostomy.

In our study, we used right subcostal incision in all cases. Cholecystectomy was performed whenever gall bladder was present as a routine. Excision of the bile duct was performed till the junction of the bile duct and pancreatic duct with the assistance of intraoperative ultrasound when doubtful. We found it helpful to not excise the gall bladder early as it may be used to exert traction on the cyst to facilitate dissection during the later part of the procedure. The drainage tube was routinely kept in the hepatorenal pouch and removed when the patient was tolerating a full oral diet. If bile leak was present, then the drain was removed after cessation of bile output through the drain for more than 48 hours.

The choice of surgery depends on the type of cyst. For type I and IVA cysts, the treatment of choice is complete excision of the cyst with a Roux-en-Y hepaticojejunostomy.

Type II cysts carry a low risk of complications, and hence it commonly agreed that cyst excision alone is adequate treatment [28]. However, in the presence of APBDU, cholecystectomy is also recommended due to the increased risk of gall bladder malignancy [20]. When the cyst is densely adherent to the underlying portal vein, it is advisable to follow Lilly's technique where the mucosa is ablated, and the portion of the adherent adventitia of the cyst on the portal vein is left behind as dissection in this setting would risk injury to the portal vein with attendant complications. [20].

Type III cysts have very low risk of malignant change; hence, excision of the cyst along with biliary tract is considered too radical. Endoscopic sphincterotomy is currently considered the treatment of choice for these patients [20].

For the extrahepatic component of type IVA cysts and for type IVB cysts, complete surgical excision of the extrahepatic biliary tree is the current standard of care. It is necessary to dissect till the confluence as intraductal membranes or septa may be a cause for stenosis and treatment failure if they are not excised circumferentially [29].

The management of intrahepatic component of type IVA cysts is still debated. Lipsett and Pitt recommend a conservative line of therapy with placement of large bore silastic transhepatic stents to enable extraction of stones in the postoperative period [18]. However, many other authors are in favor of liver resection, as it completely eradicates both extrahepatic and intrahepatic portions of the disease especially in intrahepatic cystic disease limited to the left lateral segments [20].

Type V cyst (Caroli's disease) is a rare type which is markedly difficult to manage. Management decisions are guided by the extent of intrahepatic disease (unilobar or bilobar) and the presence of chronic liver disease (congenital hepatic fibrosis, secondary biliary cirrhosis, and cholangiocarcinoma). For unilobar intrahepatic disease without liver disease, liver resection is the treatment of choice with good long-term outcomes [30]. When coexisting liver disease with portal hypertension from congenital hepatic fibrosis or secondary biliary cirrhosis is present, or if both lobes are affected, patients treated with conservative treatment develop recurrent episodes of cholangitis and variceal bleeds and eventually die from liver failure or cholangiocarcinoma. Hence, liver transplant is considered the treatment of choice for this subset of patients [20]. Transplantation represents the only option which addresses both the underlying liver disease and the CC.

We recommend excision of extrahepatic biliary tract excision for type I (all subtypes), type IV b, and the extrahepatic component of type IVa. For type II and III cysts, we recommend excision of the diverticulum-shaped cyst and endoscopic sphincterotomy, respectively. Management for intrahepatic cysts (in type IVa and V) is complex, and we recommend resection of the affected segments/lobe of liver in disease confined to one lobe of the liver. For bilobar disease and for patients with underlying cirrhosis, we recommend referral to a liver transplant unit.

### 4.2. Outcomes

Following surgery, patients need to be placed on strict lifelong surveillance as the risk of developing biliary tract malignancy is high. The risk of malignancy persists even after cyst excision. However, this risk is lower than in those in whom cyst is either not excised or residual cyst is left behind [20]. Follow-up is undertaken through physical examination, liver function tests, ultrasonography, and the tumor marker Ca 19–9 [20]. Anastomotic stricture following surgery may be treated either by percutaneous transhepatic dilatation or revision surgery [20].

## 5. Conclusion

Although choledochal cysts present commonly in children, one-fifth of choledochal cysts present in the adulthood. Hence, they should be considered in the differential diagnosis in adult patients with suspected biliary pathology. Detailed preoperative evaluation facilitates planning of surgery. Hepatobiliary surgeons in developing countries encounter many challenges due to delay in presentation, high rates of misdiagnosis, and suboptimal therapies. APBDU is uncommon in Indian patients when compared to studies on Eastern patients.

Complete surgical excision is the current standard of care for extrahepatic cyst types. For intrahepatic cystic disease, liver resection appears to offer the best outcomes if the disease is localized to one lobe or segment of the liver. For diffuse intrahepatic disease or in the presence of coexisting liver disease, transplantation offers the only chance for long-term cure. Strict long-term surveillance is advised for identification of development of cholangiocarcinoma.

## Figures and Tables

**Table 1:** Clinical presentation and associated conditions.

Symptom/associated condition	Cyst type I (28)	Cyst type IVA (*n* = 5)	Cyst type IVB (*n* = 3)	Total
Pain	27	5	3	35
Anorexia	6	3	0	9
Loss of weight	4	3	0	7
Clinical jaundice	4	2	0	6
Palpable mass	4	2	0	6
Cholangitis	4	0	1	5
Pancreatitis	1	1	0	2
Elevated serum bilirubin	8	1	0	9
Elevated serum alkaline phosphatase	9	2	0	11
Cystolithiasis with cholelithiasis	4	2	0	6
Cystolithiasis only	3	1	0	4
Cystolithiasis with hepatolithiasis	0	0	3	3
Distal biliary stricture	4	1	0	5
Perivaterian diverticulum	2	0	0	2
Liver abscess	2	0	0	2
Chronic pancreatitis	2	0	0	2
Pancreas divisum	1	0	0	1
Nonrotation of intestines	0	1	0	1

**Table 2:** Todani modification of Alonso-Lej classification.

Cyst type	Morphological description
Ia	Solitary saccular dilatation of the extrahepatic biliary system
Ib	Solitary segmental dilatation of the extrahepatic biliary system
Ic	Solitary diffuse or cylindrical dilatation of the extrahepatic biliary system
II	Diverticulum of the supraduodenal portion of the bile duct
III	Cystic dilatation of the intraduodenal portion of the bile duct (choledochocoele)
IVa	Multiple cystic dilatations involving both intrahepatic and extrahepatic biliary system
IVb	Multiple cystic dilatations involving only the extrahepatic biliary system
V	Multiple cystic dilatations involving only the intrahepatic biliary system (Caroli's disease)

**Table 3:** Operative procedures performed.

Operative procedure	Cyst type I	Cyst type IVA	Cyst type IV B
CEReYHJ	25	4	—
CEReYHJ + LLS	—	—	1
CEReYHJ + LH	—	—	2
CEReYHJ + RC	—	1	—
CEReYHJ + LP	1	1	—
CEHD	1	—	—

CEReYHJ-cyst excision with Roux-en-Y hepaticojejunostomy, LLS-left lateral segmentectomy, LH-left hepatectomy, RC-radical cholecystectomy, LP-Lilly's procedure, CEHDD-cyst excision hepaticoduodenostomy.

**Table 4:** Early complications graded by Dindo–Clavien classification.

Clavien–Dindo complication type	Number of patients
I	18
II	1
IIIB	2

**Table 5:** Late complications graded by Dindo–Clavien classification.

Clavien–Dindo complication type	Number of patients
Grade II	3
Grade IIIB	2

## Data Availability

The nominal and ordinal data used to support the findings of this study are available from the corresponding author upon request.
